# Small RNAs beyond Model Organisms: Have We Only Scratched the Surface?

**DOI:** 10.3390/ijms23084448

**Published:** 2022-04-18

**Authors:** Emilie Boutet, Samia Djerroud, Jonathan Perreault

**Affiliations:** Institut National de la Recherche Scientifique, Centre Armand-Frappier Santé Biotechnologie, Laval, QC H7V 1B7, Canada; emilie.boutet@inrs.ca (E.B.); samia.djerroud@inrs.ca (S.D.)

**Keywords:** small RNAs, non-coding RNA, genetic regulation

## Abstract

Small RNAs (sRNAs) are essential regulators in the adaptation of bacteria to environmental changes and act by binding targeted mRNAs through base complementarity. Approximately 550 distinct families of sRNAs have been identified since their initial characterization in the 1980s, accelerated by the emergence of RNA-sequencing. Small RNAs are found in a wide range of bacterial phyla, but they are more prominent in highly researched model organisms compared to the rest of the sequenced bacteria. Indeed, *Escherichia coli* and *Salmonella enterica* contain the highest number of sRNAs, with 98 and 118, respectively, with *Enterobacteriaceae* encoding 145 distinct sRNAs, while other bacteria families have only seven sRNAs on average. Although the past years brought major advances in research on sRNAs, we have perhaps only scratched the surface, even more so considering RNA annotations trail behind gene annotations. A distinctive trend can be observed for genes, whereby their number increases with genome size, but this is not observable for RNAs, although they would be expected to follow the same trend. In this perspective, we aimed at establishing a more accurate representation of the occurrence of sRNAs in bacteria, emphasizing the potential for novel sRNA discoveries.

## 1. Introduction

Small RNAs (sRNAs) are important post-transcriptional regulators involved in many cellular mechanisms such as biofilm formation, adaptation to environmental changes and virulence [1]. They modulate gene expression by base-pairing with their target mRNA either with perfect (*cis*-acting) or partial (*trans*-acting) complementarity. *Cis*-acting sRNAs (better known as antisense RNAs; asRNAs) are encoded in the opposing strand of their target mRNAs, whereas *trans*-acting sRNAs are in a different locus. The latter tend to target multiple mRNAs and often rely on the help of chaperone proteins such as Hfq or ProQ in Gram-negative bacteria [2]. Here, we focused on *trans*-acting sRNAs, though a similar analysis dedicated to asRNAs is available in the Supplementary Material (Supplementary Material Table S1–S3 and Figure S1).

The effects of sRNA binding to its mRNA target are manifold. Small RNAs are between 50 and 300 nucleotides, and they have an impact on the translation of their target mRNA, more often via downregulation of protein synthesis than upregulation [3]. The binding of an sRNA to its target can prevent the ribosome from reaching the ribosome binding site (RBS) either by directly obstructing its access or by promoting a structural change that leads to its sequestration, therefore preventing translation from occurring [4,5]. Inversely, this binding could result in changes in the secondary structure of an mRNA, releasing an RBS that would otherwise be sequestered [6]. An sRNA-Hfq complex can also promote RNA degradation by the recruitment of ribonuclease E (RNAse E) [7]. Small RNA binding can also lead to ribosome stalling, which can reveal downstream RNAse E sites and promote target mRNA degradation [8]. All this to say, sRNAs’ modes of action are diverse and rely on regulatory mechanisms that affect mRNA stability, degradation, or accessibility to the ribosome and RNA-binding proteins.

In Gram-negative bacteria, sRNAs regulation is often facilitated by chaperone proteins Hfq and ProQ. Homologs of the protein Hfq are found in approximately 50% of all sequenced bacteria [9], whereas ProQ is specific to Gram-negative microorganisms [10]. We hypothesized that sRNAs could be found in all Gram-negative bacteria encoding for either chaperone proteins. Even if it is present in Gram-positive bacteria, Hfq does not seem to operate in the same manner as in Gram-negative bacteria [10]. The identification of RNA-binding proteins in Gram-positive bacteria with a similar impact on gene regulation as Hfq and ProQ is an important missing factor in paving the way to novel sRNA discovery. It was suggested that the protein CsrA could fulfill this function in Gram-positive bacteria, but research is lacking. In fact, it was only demonstrated that CsrA could promote the interaction between the sRNA SR1 and its target in *B. subtilis* [11].

The first characterized sRNA, MicF, was described approximately 40 years ago. Initially identified as a “repressor RNA”, MicF is an sRNA that regulates an important outer membrane protein in *Escherichia coli*, OmpF [12,13,14]. Since this first breakthrough, numerous sRNAs have been identified; the rate of these discoveries has increased since the advent of next-generation sequencing, which permitted RNA-sequencing. However, their discovery mainly focused on model organisms such as *Escherichia* and *Salmonella* species, overlooking other bacteria that also have the potential to encode numerous sRNAs. We wanted to estimate whether we are far from the true number of sRNAs by getting an overview outside these common models. By demonstrating the biases toward model organisms and pathogens, we hope to pique the interest of other non-coding RNA enthusiasts and pave the way for new sRNAs discoveries.

## 2. Prevalence of sRNAs in Bacteria

Information about sRNAs annotated in bacterial genomes compiled for this article was procured from RiboGap [15] (queries are available in Supplementary Material, Table S4). This database facilitates the inspection of non-coding regions in prokaryotes. The compilation of annotated sRNAs in RiboGap comes from Rfam, a database compiling sequences from structural RNA families [16], and is limited to available annotations. However, additional sRNAs are predicted within RiboGap compared to Rfam since homology searches were executed on all prokaryotic genomes available in NCBI [17] from covariance models of the entire sRNA collection in Rfam.

Rfam allowed us to examine the prevalence of sRNAs in a wide range of bacteria, but other organism-specific databases exist. To name a few, sRNAMap is a web-based application for Gram-negative bacteria only [18], whereas sRNAdb [19] is specific to Gram-positive bacteria. RegulonDB [20] and Ecocyc [21] compile sRNAs from *E. coli*, while published data on sRNAs in Staphylococci with a focus on *Staphylococcus aureus* are gathered in the SRD database [22]. BSRD also contains a repertoire of small bacterial RNA, but most of its data are homologs found in Rfam [23]. We, therefore, chose to work with Rfam to obtain a sense of the extent of sRNAs in bacteria, but it is worth mentioning that other databases are available when the research is more focused on a particular organism, although this is generally limited to model organisms. This article also focuses on sRNAs with an E-value lower than 0.0005, to remove any sRNAs with poor homology prediction.

Since the characterization of the first sRNA in the 1980s, numerous sRNAs have been discovered in a wide range of bacterial phyla, including 549 distinct sRNA families listed in Rfam. Proteobacteria and Terrabacteria groups encode the highest number of distinct sRNAs (Table 1).

Bacteria from the phylum Proteobacteria and the Terrabacteria phylum group both encode many distinct sRNAs (345 and 210, respectively). It comes as no surprise that the Terrabacteria super-phylum group stands out from others in terms of the number of annotated sRNAs since it encompasses approximately two-thirds of all identified species, including all Gram-positive bacteria and most spore-producing bacteria [24]. It also includes human pathogens such as *Clostridium*, *Staphylococcus* and food and waterborne pathogens such as *Listeria* and *Campylobacter* [24]. Proteobacteria is a well-studied phylum since it is predominant in the human gut microbiome and often associated with multiple intestinal and extraintestinal diseases [25] and includes many human pathogens, such as those from the genera *Bordetella*, *Brucella*, *Burkholderia*, *Francisella*, *Helicobacter*, *Neisseria*, *Rickettsia*, *Salmonella* and *Yersinia* [25], which would explain incentives to study them.

**Figure 1:**
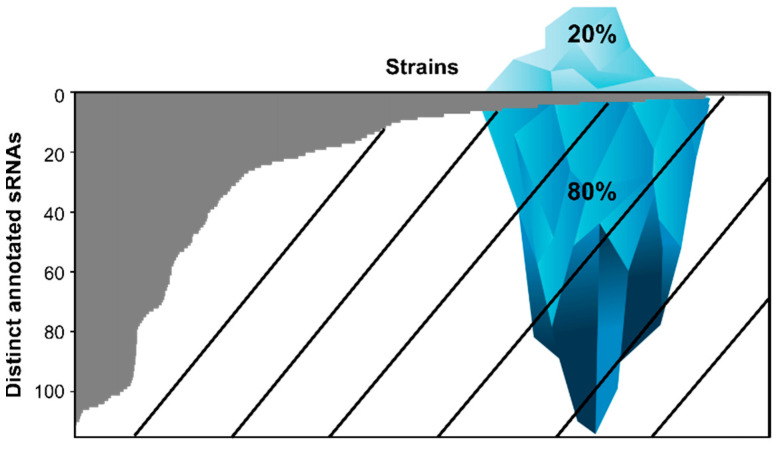
Number of distinct annotated sRNAs per bacterial strain in Proteobacteria. The iceberg is intended to be a graphical representation of the knowledge we have about the prevalence of sRNAs in Proteobacteria (gray section) as opposed to what we could be missing (hatched section). The ratio of the surface versus underwater portions of the iceberg is proportional to results represented in the graph, where the gray region is what is known (i.e., the visible part of the iceberg), and the hatched area under that region is what could be left to discover (that is, the underwater section of the iceberg). Percentages also represent this ratio. This figure represents a compilation of 2629 strains. Only sRNAs with an E-value lower than 0.0005 were considered.

Most species have a relatively small number of distinct sRNAs annotated within their genome (Supplementary Material Figure S2A), whereas those with the highest sRNA occurrences are within the phylum Proteobacteria and Terrabacteria group (Table 1). If we disregard those overrepresented phyla, the remaining bacteria have an average of only 1 to 2 sRNAs encoded in their genome (Supplementary Material Figure S2A). From that list, most are non-pathogenic and are not considered model organisms. However, there are a few exceptions, including those responsible for the sexually transmitted infections (STI), chlamydia and syphilis (*Chlamydia trachomatis* [26] and *Treponema pallidum* [27], respectively), a bacteria associated with dog bite infections (*Capnocytophaga* sp. [28]) as well as plant (*Liberibacter* sp. [29]), poultry (*Riemerella* sp. [30]) and fish (*Tenacibaculum* sp. [31]) pathogens. This list also includes model organisms in specific fields of research, such as *Chlorobaculum* sp., which is used to study sulfur metabolism and photosynthesis [32], as well as *Porphyromonas* sp., used to study the interaction of anaerobic bacteria with host cells [33]. Despite their relevance as pathogens and in fundamental research, the presence of sRNAs has not been examined in these species. A genome-wide transcriptomic study was realized in *Chlamydia trachomatis,* identifying 43 candidate sRNAs [34], but only one is referenced within the Rfam database, IhtA [35]. It would be interesting to dedicate future sRNA studies to these bacteria since they have a very small number of annotated sRNAs. Conversely, numerous bacterial strains from the major Gram-negative phylum Proteobacteria encode for large numbers of sRNAs (Figure 1).

Figure 1 represents the potential to discover novel sRNAs, where the underwater portion of the iceberg depicts the sRNAs that remain to be found if all strains contain similar quantities of sRNAs as the most well-studied bacteria. Given chaperone proteins ProQ and Hfq are highly conserved in Gram-negative bacteria [36,37], we feel comfortable making this extrapolation since the occurrence of either or both chaperone proteins in the genome of bacteria could be a good indication of the presence of sRNAs. We also represented the potential for sRNA discovery in bacteria from other phyla (Supplementary Material Figure S2). However, given the chaperone protein ProQ is absent in Gram-positive bacteria [37], this extrapolation is less reliable. Despite the fact that an Hfq homolog is present in Gram-positive bacteria, it does not seem to act as a matchmaker for sRNAs and their targets, which is its most prominent role in Gram-negative bacteria [1].

### 2.1. Species Encoding for sRNAs

**Figure 2:**
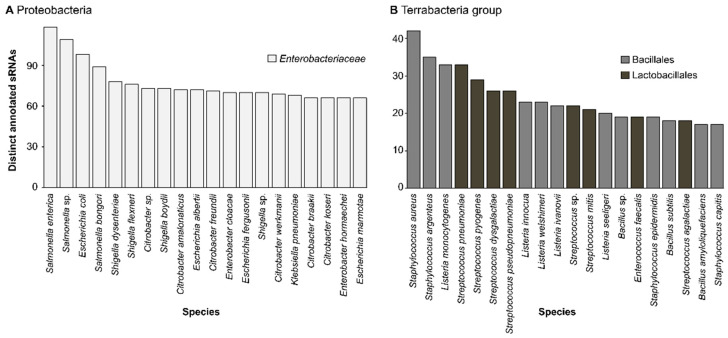
Top 20 bacterial species with the highest number of distinct annotated sRNAs in (**A**) Proteobacteria and in (**B**) bacteria from the Terrabacteria group. Species denoted with “sp.” represent instances where only the genus of the bacteria was noted. It can be observed that in (**A**), all species are from the same family, *Enterobacteriaceae*. In (**B**), species from different orders are emphasized by their own color. The number of distinct sRNAs considers all strains for each species. Only sRNAs with an E-value lower than 0.0005 were considered.

The model organisms *Salmonella enterica* and *Escherichia coli* contain the most distinct sRNAs annotated in their genome, with 118 and 98, respectively, if you consider all strains for each species (Figure 2).

For Proteobacteria, it is hardly surprising that *Escherichia coli* is at the top of the list since it is the microbiologist’s bacteria of choice in the laboratory due to its ease of handling and the availability of associated tools. It is the most studied and best-understood bacteria [38], and much of our fundamental understanding of biology has come from this model organism, including the genetic code [39] and the characterization of the first sRNA [12,13,14]. As a very close parent of *E. coli*, *Salmonella enterica* is expected to contain similar sRNAs, although many other species-specific sRNAs were found, presumably due to extensive research on host-pathogen interactions, which made use of this model organism. *Salmonella* sp. are attractive model organisms because they can target a wide range of hosts with multiple evasion strategies giving an idea of major tactics adopted by other pathogens [40]. For example, the sRNA IsrJ in *Salmonella* sp. was demonstrated to encourage the invasion of epithelial cells, and knockout strains for this sRNA lead to less invasive mutants [41]. For Proteobacteria, all the bacteria from the figure belong to the family *Enterobacteriaceae*, which encodes for 145 distinct sRNAs compared to an average of seven for all other bacterial families.

In the case of bacteria from the Terrabacteria group, human pathogens *Staphylococcus* and *Listeria* have the highest number of distinct annotated sRNAs [42,43]. As for *Streptococcus* sp., some of its species are considered part of the normal human microbiome, but others, such as *Streptococcus pneumonia,* are responsible for most cases of pneumonia worldwide [44]. The model organism *Bacillus subtilis* also has a high number of annotated sRNAs, perhaps because it is a common Gram-positive bacteria to investigate biofilm formation [45], among other processes. As we can observe, the species with the most annotated sRNAs are those associated with high research intensity, either because they are a threat to human health or due to their attractiveness as model organisms (Table 2). By digging past this bacterium all-star list, we hypothesized that multiple novel sRNAs are left to be discovered. By focusing on less standard organisms, we could potentially extend the role of sRNAs to unexpected new functions. Moreover, sRNAs discovered in understudied bacteria could be the missing puzzle piece to solve an incomplete regulatory mechanism in a model organism.

### 2.2. Most Abundant Small RNAs

**Figure 3:**
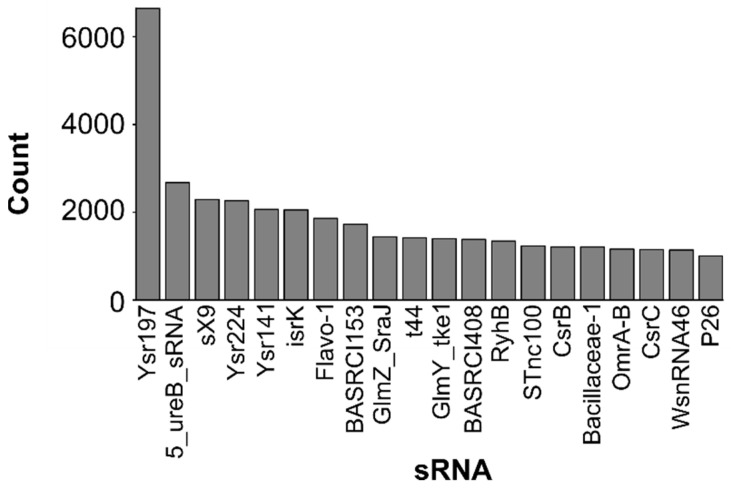
Top 20 sRNAs annotated in bacteria. Each individual occurrence of sRNAs were counted, even if some were found multiple times within the same genome. Only sRNAs with an E-value lower than 0.0005 were taken into consideration.

We were then interested to know which sRNAs were the most present throughout all bacterial genomes. If an sRNA was annotated multiple times within the same strain, we counted all individual instances (Figure 3).

From the top lists of sRNAs, most were discovered in human pathogenic bacteria (Ysr197 [51,52], 5_ureB_sRNA [53], Ysr224 [52,54], Ysr141 [55], isrK [41,56], BASRCI153 [57], BASRCI408 [57] and STnc100 [56]), in causative agent of plant infection (sX9 [58]) or in parasitic microbes (WsnRNA-46 [59]). The latter was found in *Wolbachia* sp., the most prevailing vertically transmitted endosymbiont around the world, impacting more than 40% of arthropods [59]. The remaining were found in the model organism *E. coli* (GlmZ_SraJ [54,60,61,62], t44 [63], GlmY_tke1 [54,60,61,62], RyhB [64,65,66,67,68,69,70], CsrB [54,71,72,73,74,75,76,77], OmrA-B [64,78,79,80] and CsrC [64,81]) or by computational homology searches (Flavo-1 [82], Bacillaceae-1 [82] and P26 [83]) (Table 3).

In other words, not only are we missing numerous sRNA instances in various bacteria, as underscored by Figure 1, but the diversity of sRNA families is also expected to be much greater. Indeed, most sRNAs are unique to limited taxonomic groups, which means that each exploratory sRNA study in an underrepresented taxon will likely lead to the discovery of novel sRNA families. Then, by homology searches, they could be related to other bacteria of interest and further deepen our knowledge of gene regulation mediated by sRNAs.

## 3. Biases towards Model Organisms and Pathogens

**Figure 4:**
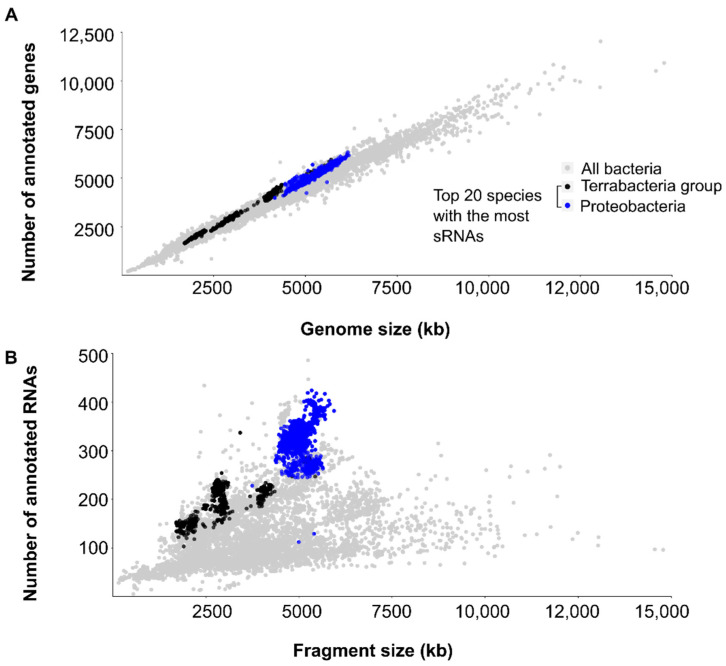
Number of annotated genes and RNAs in bacteria. Data required for the creation of this graph were taken from RiboGap [15]. (**A**) The number of annotated genes is graphed according to the genome size, which comprises all chromosomes and plasmids of each individual strain if applicable. (**B**) The number of annotated RNAs is graphed according to the “fragment size”, which considers chromosomes and plasmids separately for each individual strain. RNAs are not limited only to sRNAs but also include CRISPR RNAs, antisense RNAs, sRNAs, long non-coding RNAs (lncRNAs), rRNAs, ribozymes, tRNAs and cis-regulatory elements. Species from Terrabacteria group and Proteobacteria that were found to have the most annotated sRNAs (Figure 2) are represented by black and blue dots, respectively; all other strains are shown in gray.

In order to demonstrate that research intensity is biased toward model organisms, pathogens, and closely related species, we looked at the number of annotated genes and RNAs in bacteria (Figure 4).

Information about genome size and the number of annotated genes and RNA comes from RiboGap [15], which extracts data from the NCBI FTP site [17]. For gene annotations, sizes are based on complete genomes, which include all plasmids and chromosomes of a given strain if applicable. However, RNAs are compiled per “DNA fragment” (chromosome or plasmid) since it is not accessible per genome within the RiboGap database. The size of each fragment was taken from all available Genbank files from the NCBI FTP site [17]. Results were limited by the available annotations. For example, some strains did not have annotated genes in NCBI and were removed from Figure 4. Moreover, some entries were mislabeled as complete genomes but were, in fact, WGS (Whole Genome Shotguns) projects with incomplete genomes, leading to a miscalculation in the number of genes (values doubled up). These erroneous data were removed from Figure 4 (shown for transparency purposes in Supplementary Material Figure S3).

Expectedly, the number of annotated genes increases proportionally with the genome size, with on average one gene per kb and a relative standard deviation (RSD) of 7% (Figure 4A). The top species with the most annotated sRNAs (Figure 2) from Proteobacteria and Terrabacteria groups (blue and black dot, respectively, Figure 4A) tend to have slightly higher numbers of genes for a given genome length. We also graphed the number of annotated RNAs compared to the fragment size, which highlights the disparity in the annotation of RNA versus protein-coding genes. There is, on average, one annotated RNA every 25 kb with a relative standard deviation of 47%, emphasizing how spread out the values are from the average number, ranging from ~1/10 kb to ~1/100 kb (Figure 4B). Information about RNA families comes from RiboGap [15] and is derived from Rfam (except for terminators, which can be found in RiboGap but were not included in these results). In principle, we should expect a similar trend for RNAs (Figure 4B) as for genes (Figure 4A), i.e., the number of annotated RNAs should increase proportionally with fragment size. However, it is clearly not the case here, emphasizing how RNA annotations trail behind gene annotations.

Annotations are dependent on the research intensity associated with each strain: the fact that some RNAs are not annotated does not mean that they are not present, but simply that they have yet to be identified. When we emphasize the species with the most annotated sRNAs in their genome (black and blue dots, Figure 4B), they also tend to be those that have the highest number of RNAs in general for a given fragment length. Therefore, their large number of annotated sRNAs likely results from high research intensity. We recreated Figure 4, this time emphasizing bacteria labeled as human pathogens in RiboGap [15] (Supplementary Material Figure S4). Even if there are large incentives to study human pathogenic bacteria, only a handful of model organisms were well characterized. There is still room for novel RNAs discovery even among numerous pathogens, as suggested by the fact that the number of annotated RNAs does not necessarily increase as expected with fragment size.

## 4. Conclusions and Perspectives

Small RNAs are important for gene regulation and modulation of responses to environmental changes. They are found in numerous bacterial phyla, especially Proteobacteria and Terrabacteria groups. However, we underestimate their prevalence because of the focus on model organisms and pathogens. Genera encoding for the highest number of sRNAs are human pathogens (*Salmonella*, *Escherichia*, *Citrobacter*, *Shigella*, *Enterobacter*, *Klebsiella*, *Streptococcus*, *Staphylococcus* and *Listeria,* amongst others) or model organisms (*Bacillus*, *Escherichia*, *Salmonella* and others). Only a small fraction of all bacteria encode for numerous sRNAs, but it would be surprising that others would not have the same variety of regulatory RNAs, especially if they encode for the RNA chaperone proteins Hfq and/or ProQ. Moreover, the diversity of sRNAs is anticipated to be much greater since most sRNAs are unique to limited taxonomic groups. For instance, the species that encode the most distinct sRNAs within the phylum Proteobacteria are all from the same family, *Enterobacteriaceae*.

Expectedly, species associated with high research intensity are also those with the largest number of annotated genes (relative to genome size) and even more so of RNAs, but what was less obvious before is how much RNA annotations fall behind gene annotations. By increasing RNA studies of infrequently studied bacteria, we could improve our capacity to annotate sRNAs and our knowledge of the extent of RNA families in bacteria, including sRNAs.

Even if there is still much to learn on sRNAs in major experimental models, our goal was to highlight the potential to discover novel sRNAs by stressing that current findings are focused on model organisms and pathogens. It was also an opportunity to take stock of the extent of our knowledge. Although there are fewer incentives to study bacteria that are neither models nor pathogens nor of direct industrial interest, new sRNA discoveries could deepen our comprehension of genetic regulation and perhaps lead to new and fascinating mechanisms. Furthermore, beyond the *E. coli* and *B. subtilis* models, there are numerous organisms that provide important models for specific biological processes. A few examples include *Methylorubrum extorquens* for the metabolism of 1-carbon compounds [85], *Myxococcus xanthus* for bacterial social behavior [86], *Azotobacter vinelandii* for nitrogen fixation [87] or *Mycoplasma genitalium* for minimal organisms [88]. RNA-seq and sRNA discovery methodologies permitted transcriptome-wide evaluation of potential sRNAs, even if further experimental validation requires a significant amount of work. Small RNAs should still be in the spotlight of research in relation to non-coding RNA-mediated genetic regulation because we have just scratched the surface of their full potential and likely have an underappreciation of the true complexity of the regulation of gene expression by sRNAs in bacteria.

## Figures and Tables

**Table 1:** Number of distinct annotated sRNAs in different phylum.

Phylum Group	sRNAs
Acidobacteria	4
Aquificae	1
Calditrichaeota	1
Dictyoglomi	1
FCB group ^1^	16
Fusobacteria	2
Nitrospirae	3
PVC group ^2^	8
Proteobacteria	345
Spirochaetes	6
Synergistetes	1
Terrabacteria group	210
Thermodesulfobacteria	1
Thermotogae	1

^1^ FCB group stands for Fibrobacteres, Chlorobi, and Bacteroidetes, whereas ^2^ PVC group represents Planctomycetes, Verrucomicrobia, and Chlamydiae.

**Table 2:** Description of genus encoding for the most distinct sRNAs.

Genus	Nb of Distinct sRNAs ^1^	Description	Ref
Proteobacteria			
*Salmonella*	119	Model organism to study host-pathogen interactions	[40]
*Escherichia*	99	Most well-understood bacteria	[38]
*Citrobacter*	88	Third most common urinary pathogen	[46]
*Shigella*	85	Causative pathogen of shigellosis	[47]
*Enterobacter*	78	Responsible for nosocomial infections	[48]
*Klebsiella*	74	Nosocomial pathogen, model organism to study drug resistance	[49]
Terrabacteria group			
*Streptococcus*	55	Responsible for most cases of pneumonia worldwide	[44]
*Staphylococcus*	46	Most prevalent cause of infection in hospitalized patient	[42]
*Listeria*	35	Foodborne human pathogens causing central nervous system infections	[43]
*Bacillus*	26	Most-studied Gram-positive bacteria, model organisms for cellular development	[45]
*Enterococcus*	25	Principal cause of the healthcare-associated death worldwide	[48,50]

^1^ The number represents the quantity of distinct annotated sRNAs in all bacterial strains within this genus. Only sRNAs with a E-value lower than 0.0005 were considered.

**Table 3:** Description of top 20 most prevalent sRNAs in bacteria.

sRNA	Description	Rfam ID	sRNA Expression	Discovered in	Ref
Ysr197	*Yersinia* sRNA 197	RF02849	Expressed in exponential phase	*Yersinia pseudotuberculosis*	[52]
5_ureB_sRNA	-	RF02514	Downregulate expression of operon *ureAB*	*Helicobacter pylori*	[84]
sX9	*Xanthomonas* sRNA sX9	RF02228	-	*Xanthomonas campestris pv*. vesicatoria (Xcv)	[58]
Ysr224	*Yersinia* sRNA 224	RF02770	Temperature-responsive	*Yersinia pseudotuberculosis*	[52,54]
Ysr141	*Yersinia* sRNA 141	RF02675	Influence the expression of Yop-Ysc type III secretion system (T3SS) (critical system for virulence)	*Yersinia pestis*	[55]
isrK	isrK Hfq binding RNA	RF01394	Stationary phase, low oxygen, low magnesium	*Salmonella typhimurium*	[41,56]
Flavo-1	-	RF01705	-	Bacteroidetes	[82]
BASRCI153	*Brucella* sRNA CI153	RF02604	Putative target: BAB1_1361	*Brucella abortus*	[57]
GlmZ_SraJ	GlmZ RNA activator of *glmS* mRNA	RF00083	activator of *glmS* mRNA	*Escherichia coli*	[54,60,61,62]
t44	-	RF00127	-	*Escherichia coli*	[63]
GlmY_tke1	GlmZ RNA activator of *glmS* mRNA	RF00128	activator of *glmS* mRNA	*Escherichia coli*	[54,60,61,62]
BASRCI408	*Brucella* sRNA CI408	RF02599	Putative target: BAB1_2002	*Brucella abortus*	[57]
RyhB	-	RF00057	Iron metabolism [67], regulates siderophore production and virulence [69], persistence regulation [70]	*Escherichia coli*	[64,65,66,67,68,69,70]
STnc100	Gammaproteobacterial sRNA STnc100	RF02076	-	*Salmonella* sp.	[56]
CsrB	CsrB/RsmB RNA family	RF00018	Binds the CrsA protein	*Escherichia coli*	[54,71,72,73,74,75,76,77]
Bacillaceae-1	-	RF01690	-	Bacteroidetes	[82]
OmrA-B	-	RF00079	Target several genes encoding outer membrane proteins	*Escherichia coli*	[64,78,79,80]
CsrC	-	RF00084	Binds the CrsA protein	*Escherichia coli*	[64,81]
Ysr276	*Yersinia* sRNA 276	RF02850	-	*Yersinia pseudotuberculosis*	[52]
WsnRNA46	*Wolbachia* sRNA 46	RF02625	Expressed in cells infected by parasitic microbe *Wolbachia*	*Wolbachia* sp.	[59]
P26	*Pseudomonas* sRNA P26	RF00630	-	*Pseudomonas aeruginosa*	[83]

## Data Availability

Data are contained within the article or Supplementary Materials.

## Supplementary Materials

The following supporting information can be downloaded at: https://www.mdpi.com/article/10.3390/ijms23084448/s1. A separate analysis was done for asRNAs [15,16,17,38,40,46,47,48,49,82,89,90,91,92,93,94,95,96,97,98,99,100,101,102,103,104,105,106,107,108,109,110,111,112,113,114,115].
