# Supplementary material for: Small RNAs beyond Model Organisms: Have We Only Scratched the Surface?
Source: Int J Mol Sci. 2022 Apr 18;23(8):4448. doi: 10.3390/ijms23084448 (PMC9029176; doi:10.3390/ijms23084448)
Supplement: Supplementary file 1 [file ijms-23-04448-s001.zip › ijms-1667467-supplementary.pdf]

Supplementary material for: Small RNAs beyond model organisms; have we only scratched the surface?

Emilie Boutet [1], Samia Djerroud [1], and Jonathan Perreault [1,] *

[1] INRS- Centre Armand-Frappier Santé Biotechnologie; emilie.boutet@inrs.ca (E.B.); jonathan.perreault@inrs.ca (J.P.)
* Correspondence: Jonathan.perreault@inrs.ca

Table of Contents

1. Antisense RNA 1
   1.1 Prevalence of asRNAs in bacteria 2
2. Materials and Methods 5
   2.1 RiboGap 5
   2.2 Graphical representation 5
3. Supplementary Figures 6
4. References 8

1. Antisense RNA

Antisense RNAs play an essential role in genetic regulation and are associated in the tight regulation of transposase, toxic proteins, transcription regulators and virulence proteins to name a few (reviewed in [[1]]). They bind with their targeted mRNA with perfect complementarity since they are encoded in the opposite strand. Their dimension varies greatly, from a few hundred nucleotides (100 to 300 nt) to larger sizes (700 to 3500 nt) [[2]]. The formation of an asRNA-mRNA complex could impact the secondary structure of both RNAs, leading to a change in their stability and perhaps degradation [[3]]. The binding of asRNA to its target could prevent the ribosome from reaching the RBS. Antisense sRNA could also impact the mRNA in the opposite strand due to transcription interference without directly binding with one another. Divergently transcribing promoters could interfere with each other, resulting in the collision of RNA polymerase complexes for example [[2]]. An elongating RNA polymerase (RNAP) on the antisense strand could impede the formation of an initiation complex on the sense strand through transcription occlusion or dislodged an already formed one through sitting duck interference [[2]].

Information about asRNAs comes from Ribogap [[4]], which extracts data from Rfam to facilitate the analysis of non-coding RNA [[5]]. Only asRNAs with an E-value lower than 0.0005 were taken into consideration. Rfam is a database on RNA families bases on secondary structures and covariance model. Since asRNAs do not rely on secondary structures, they may be underrepresented in Rfam. Nevertheless, it still gives us a good estimation of the extent of knowledge of asRNAs in bacteria. Moreover, we are limited by the available annotations in Rfam. For example, the sRNA MicF is known to be encoded in a different locus than its target, the outer membrane protein OmpF in *Escherichia coli [[6]].* It would therefore meet criteria to be classified as a trans-acting sRNA rather than an asRNA. However, early research did not make the same distinction between asRNA and sRNA, so it was classified as an asRNA in Rfam, a categorization which remains. It would be tedious to go through the list of all asRNAs family in Rfam to verify their classification, and we are confident that it would not change the conclusion of this perspective article.

1.1. Prevalence of asRNAs in bacteria

Forty distinct asRNAs were annotated in bacterial genomes based on the Rfam database. Like for sRNAs, the phyla Proteobacteria and those from the Terrabacteria group also encode for the most distinct asRNAs (Table S1), with 29 and 17 respectively. Interestingly, the proportion of asRNAs in the two most studied phyla relative to the sum of all phyla from Table S1 is similar to that of sRNAs from Table 1 (60% vs. 58% for Proteobacteria and 35% vs. 35% for Terrabacteria), even though asRNAs act through very different mechanisms, also suggesting that these numbers strongly correlate with the "intensity of research" within these phyla.

Table S1. Number of distinct annotated asRNAs encoded in different phylum.

Phylum group	asRNAs
FCB group [1]	2
Proteobacteria	29
Terrabacteria group	17

[1] FCB group stands for Fibrobacteres, Chlorobi, and Bacteroidetes, whereas [2] PVC group represents Planctomycetes, Verrucomicrobia, and Chlamydiae.

Genus that encodes for the most distinct asRNAs are all from the family *Enterobacteriaceae*, apart for *Serratia marcescens* that is a *Yersiniaceae* (Figure S1, A). We also in examined the top 10 most annotated asRNAs in all bacteria (Figure S1, B).

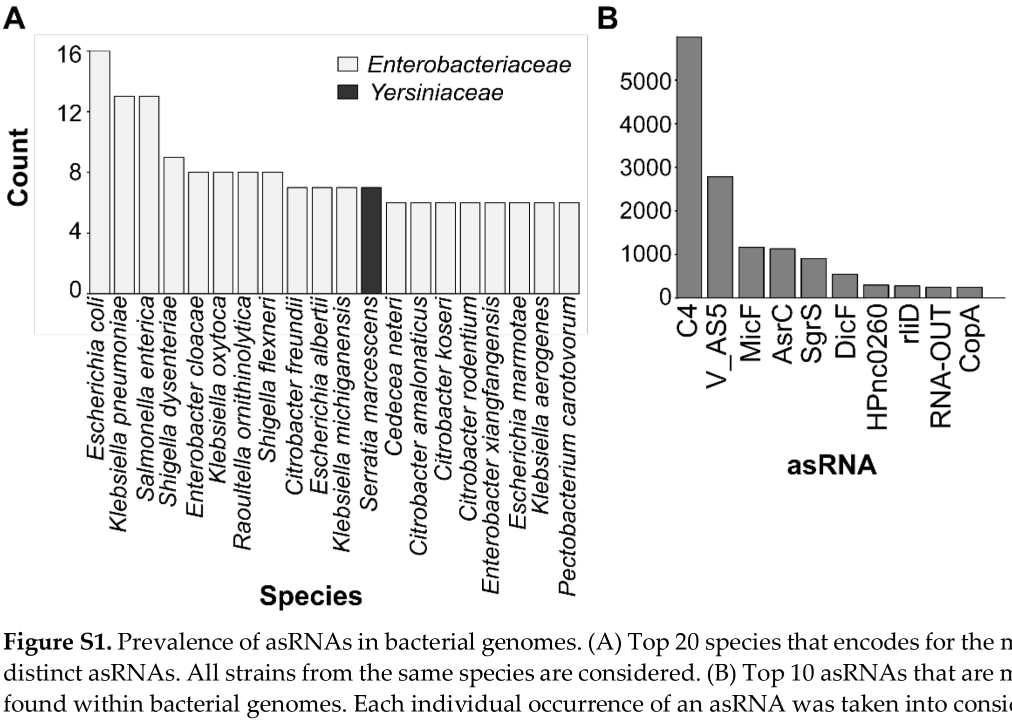

Figure S1. Prevalence of asRNAs in bacterial genomes. (A) Top 20 species that encodes for the most distinct asRNAs. All strains from the same species are considered. (B) Top 10 asRNAs that are most found within bacterial genomes. Each individual occurrence of an asRNA was taken into consideration. Only asRNAs with E-value lower than 0.0005 were kept.

Some of the genera that encode for the highest number of distinct asRNAs were also those that contain the most sRNAs. However, three genera were not discussed before: *Raoultella*, *Serratia* and *Cedecea* (Table S2, in bold) and they are all human pathogens. *Cedecea neteri* for example was isolated at the CDC (Centers for Disease Control and Prevention), from where its name originates. Even if the incidence of *Cedecea* infections is infrequent, increasing occurrences and its antibiotic resistance warrant more research interest. It is considered an opportunistic pathogen, since it is isolated in immunocompromised patients [7].

Table S2. Description of genus encoding for the most distinct asRNAs.

Genus	Nb of distinct asRNAs[1]	Description	Ref
Escherichia	16	Most well-understood bacteria	8
Klebsiella	13	Nosocomial pathogen, model organism to study drug resistance	9
Salmonella	13	Model organism to study host-pathogen interactions	10
Shigella	11	Causative pathogen of shigellosis	11
Enterobacter	9	Responsible for nosocomial infections	12
Citrobacter	9	Third most common urinary pathogen	13
Raoultella	8	Associated with histamine poisoning in human	14
Serratia	8	Opportunistic nosocomial pathogen	15
Cedecea	6	Rare pathogen associated with urinary tract infections; antibiotic resistance	16

[1]The number represents the quantity of distinct sRNAs in all bacterial strains within this genus.

The most annotated asRNAs in bacterial genome were also discovered in the model organism *E. coli (*MicF [6], SgrS [17-19], DicF [20-23] and RNA-OUT [24]), in pathogens (V_AS5 [25], AsrC [26], HPnc0260 [27], rliD [28] and CopA [29-32]) or as part of computational homology searches (C4 [33]) (Table S3).

Table S3. Description of top 10 most prevalent asRNAs in bacteria.

asRNA	Description	RFAM	asRNA expression	Discovered in	Ref
C4	C4 antisense RNA	RF01695	-	Proteobacteria, Phages	33
V_AS5	*Vibrio* RNA AS5	RF02818	-	*Vibrio cholerae*	25
MicF	-	RF00033	Regulates outer membrane protein OmpF	*Escherichia coli*	6
AsrC	antisense RNA of *rseC* mRNA	RF02746	Target *rseC*; promote bacterial motility	*Salmonella enterica* serovar typhi	26
SgrS	-	RF00534	Coordinate response to glucose-phosphate stress	*Escherichia coli*	17-19
DicF	-	RF00039	Inhibitor of gene *ftsZ* involved in cell division	*Escherichia coli*	20-23
HPnc0260	Bacterial antisense RNA HPnc0260	RF02194	-	*Helicobacter pylori*	27
rliD	*Listeria* sRNA *rliD*	RF01494	Antisense of the gene *pnpA*, a Polynucleotide phosphorylase	*Listeria monocytogenes*	28
RNA-OUT	-	RF00240	Tn10/IS10 antisense system	*Escherichia coli*	24
CopA	CopA-like RNA	RF00042	Regulate copy number of plasmid R1	Plasmid R1 (first isolated from *Salmonella* sp. *[34]*	29-32

As demonstrated, most of the knowledge we have for asRNAs comes from study on research-intensive pathogens and model organisms. The most prevalent asRNAs are also found in closely related species of the same order, almost exclusively from the *Enterobacteriaceae* family, apart from *Yersiniaceae*. By extending our research to other bacteria, we could improve our understanding of the role of asRNAs in genetic regulation.

2. Materials and Methods

2.1. RiboGap

Information about sRNA, coding sequence and annotations was extracted from RiboGap [4]. This database is accessible via a web interface: http://ribogap.iaf.inrs.ca/ribo_gap_advanced_version_ribogap_v2.pl (version 2). Queries can be selected with a user-friendly interface or be typed in SQL directly in the appropriate box. Queries used for this article are found in Table S4. All genetic information (RNAs and genes) compiled for each species are naturally found in their genome.

Table S4. RiboGap queries.

	Query
sRNAs annotated in bacteria	select distinct fragment.taxonomy,fragment.description as description_of_fragment,rna_family.fam_id,rna_family.fam_name,rna_family.description as description_of_rna_family,rna_family.type,rna_known.evalue from fragment inner join rna_known on fragment.fragment = rna_known.fragment inner join rna_family on rna_known.fam_id = rna_family.fam_id where rna_family.description Like '%sRNA%' OR rna_family.type Like '%sRNA%' LIMIT 0, 50
Bacterial genome size and number of annotated genes	select distinct fragment.fragment,fragment.length,fragment.chromosome,fragment.gene_num,fragment.description from fragment where fragment.chromosome Like '%chromosome%' LIMIT 0, 50
Number of annotated RNA	select * from fragment inner join rna_known on fragment.fragment=rna_known.fragment where fragment.chromosome like '%chromosome%' and fam_id like '%RF%';
Number of annotated tRNA	select * from fragment inner join rna_known on fragment.fragment=rna_known.fragment where fragment.chromosome like '%chromosome%' and fam_id like '%tRNA%';
AsRNAs annotated in bacteria	select distinct fragment.taxonomy,fragment.description as description_of_fragment,gap5.accession,rna_family.fam_id,rna_family.fam_name,rna_family.description as description_of_rna_family,rna_family.type,rna_known.evalue from fragment inner join gap5 on fragment.fragment = gap5.fragment inner join rna_gap5 on gap5.num_cle = rna_gap5.num_cle inner join rna_known on rna_gap5.rna_id = rna_known.rna_id inner join rna_family on rna_known.fam_id = rna_family.fam_id where rna_family.type Like '%antisense%' OR rna_family.type Like '%asRNA%' LIMIT 0, 50
Human pathogenic bacteria	select distinct fragment.fragment,fragment.description,organism.pathogenic_in from organism INNER JOIN fragment on organism.organism_id=fragment.organism_id where organism.pathogenic_in Like '%human%' LIMIT 0, 50

Only RNAs with an E-value lower than 0.0005 were kept for this article. We made similar queries that would consider E-values up to 100, which increased the number of sRNAs by ~20% in some well-studied classes and almost doubled number of sRNAs in less studied classes, which does not fundamentally change our conclusions (even if it had significantly changed our figures), but would, however, have resulted in a much less reliable set of data to prepare figures and tables presented in this article. Size of fragments (chromosome or plasmid) is not available yet on Ribogap. This information was therefore extracted from all available Genbank files in the FTP of NCBI [35].

2.2. Graphical representation

Graphic representations were created with the ggplot2 package [36] within Jupyter notebook [37].

3. Supplementary Figures

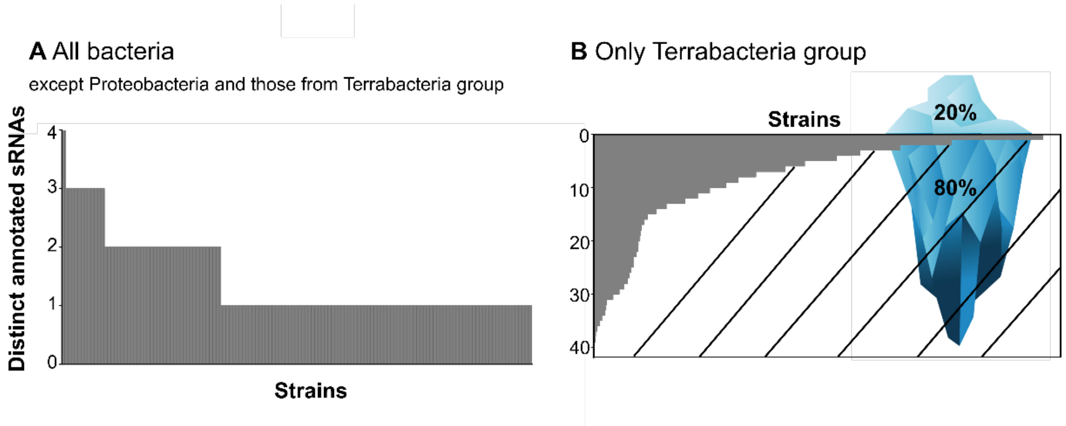

Figure S2. Number of distinct annotated sRNAs in (A) all bacteria except Proteobacteria and those from the Terrabacteria group and in (B) only bacteria from the latter. The iceberg is a representation of the number of sRNAs that we could be missing in the Terrabacteria group, where the above water portion of the iceberg portrays the already known sRNAs (gray section), and the underwater section depicts what could be left to be discovered (hatched section) if all bacteria contained as many sRNAs as those with the highest number of them. Percentages also represent this ratio of what is known versus what could be left to discover. This figure represents a compilation of 398 and 1604 strains in (A) and (B) respectively.

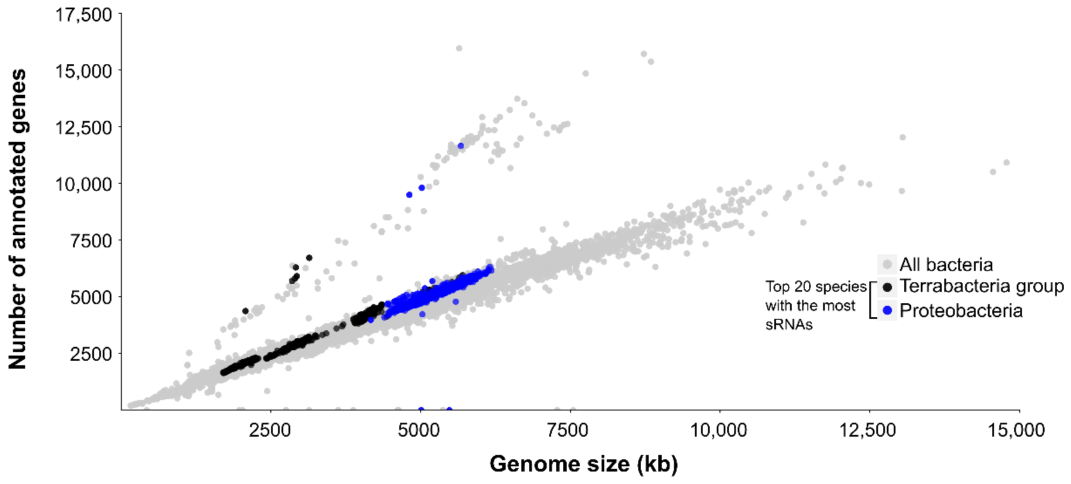

Figure S3. Number of annotated genes compared to genome size. The top 20 species containing the most annotated sRNAs from Terrabacteria group and Proteobacteria are emphasized with black and blue dots respectively. This figure also includes outliers from bacterial strains with no annotations available in NCBI [35] and some mislabeled as complete genomes.

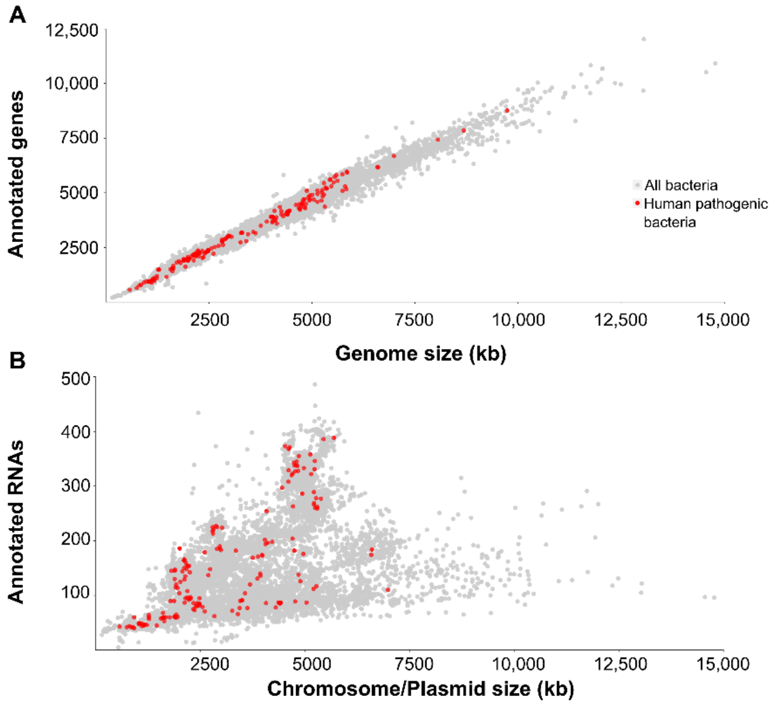

Figure S4. Number of annotated genes and RNA, where human pathogenic bacteria are emphasized in red. (A) Number of annotated genes compared to genome size (all chromosomes and plasmids for each bacterial strain are considered when applicable). (B) Number of annotated RNAs compared to fragment size (chromosomes or plasmids). RNAs include CRISPR RNAs, antisense RNAs, sRNAs, tRNAs, long non-coding RNAs, ribozymes and cis-regulatory elements. Bacteria were considered as human pathogens when they were labeled as such within the RiboGap database [4] and are derived from former tables that NCBI does not update anymore and thus do not include all pathogens (it includes 217 pathogens from a sample of 1023 bacteria and can thus still be considered a substantial sample).

From the analysis shown in Figure S4, it appears clear that even if most of the species with the highest number of annotated sRNAs (and ncRNAs in general) are pathogens, even among human pathogens there are still numerous bacteria that are understudied from the point of view of their ncRNAs.

References

1. Thomason MK, Storz G. Bacterial antisense RNAs: how many are there, and what are they doing? Annual review of genetics 2010; 44:167-88.
2. Georg J, Hess WR. cis-antisense RNA, another level of gene regulation in bacteria. Microbiology and Molecular Biology Reviews 2011; 75:286-300.
3. Dühring U, Axmann IM, Hess WR, Wilde A. An internal antisense RNA regulates expression of the photosynthesis gene isiA. Proceedings of the National Academy of Sciences 2006; 103:7054-8.
4. Naghdi MR, Smail K, Wang JX, Wade F, Breaker RR, Perreault J. Search for 5′-leader regulatory RNA structures based on gene annotation aided by the RiboGap database. Methods 2017; 117:3-13.
5. Kalvari I, Nawrocki EP, Ontiveros-Palacios N, Argasinska J, Lamkiewicz K, Marz M, et al. Rfam 14: expanded coverage of metagenomic, viral and microRNA families. Nucleic Acids Research 2021; 49:D192-D200.
6. Delihas N, Forst S. MicF: an antisense RNA gene involved in response of Escherichia coli to global stress factors. Journal of molecular biology 2001; 313:1-12.
7. Thompson DK, Sharkady SM. Expanding spectrum of opportunistic Cedecea infections: Current clinical status and multidrug resistance. International Journal of Infectious Diseases 2020; 100:461-9.
8. Blount ZD. The natural history of model organisms: The unexhausted potential of E. coli. Elife 2015; 4:e05826.
9. Bi D, Jiang X, Sheng Z-K, Ngmenterebo D, Tai C, Wang M, et al. Mapping the resistance-associated mobilome of a carbapenem-resistant Klebsiella pneumoniae strain reveals insights into factors shaping these regions and facilitates generation of a 'resistance-disarmed' model organism. Journal of Antimicrobial Chemotherapy 2015; 70:2770-4.

10. Garai P, Gnanadhas DP, Chakravortty D. Salmonella enterica serovars Typhimurium and Typhi as model organisms: revealing paradigm of host-pathogen interactions. Virulence 2012; 3:377-88.
11. Killackey SA, Sorbara MT, Girardin SE. Cellular aspects of Shigella pathogenesis: focus on the manipulation of host cell processes. Frontiers in cellular and infection microbiology 2016; 6:38.
12. Sanders Jr WE, Sanders CC. Enterobacter spp.: pathogens poised to flourish at the turn of the century. Clinical microbiology reviews 1997; 10:220-41.
13. Ranjan K, Ranjan N. Citrobacter: An emerging health care associated urinary pathogen. Urology annals 2013; 5:313.
14. Hajjar R, Ambaraghassi G, Sebajang H, Schwenter F, Su S-H. Raoultella ornithinolytica: emergence and resistance. Infection and Drug Resistance 2020; 13:1091.
15. Khanna A, Khanna M, Aggarwal A. Serratia marcescens-a rare opportunistic nosocomial pathogen and measures to limit its spread in hospitalized patients. Journal of clinical and diagnostic research: JCDR 2013; 7:243.
16. Ahmad H, Masroor T, Parmar SA, Panigrahi D. Urinary tract infection by a rare pathogen Cedecea neteri in a pregnant female with Polyhydramnios: rare case report from UAE. BMC Infectious Diseases 2021; 21:1-6.
17. Aiba H. Mechanism of RNA silencing by Hfq-binding small RNAs. Current opinion in microbiology 2007; 10:134-9.
18. Vanderpool CK. Physiological consequences of small RNA-mediated regulation of glucose-phosphate stress. Current opinion in microbiology 2007; 10:146-51.
19. Vanderpool CK, Gottesman S. The novel transcription factor SgrR coordinates the response to glucose-phosphate stress. Journal of bacteriology 2007; 189:2238-48.
20. Bouché F, Bouché JP. Genetic evidence that DicF, a second division inhibitor encoded by the Escherichia coli dicB operon, is probably RNA. Molecular microbiology 1989; 3:991-4.
21. Faubladier M, Bouché J-P. Division inhibition gene dicF of Escherichia coli reveals a widespread group of prophage sequences in bacterial genomes. Journal of bacteriology 1994; 176:1150-6.
22. Murashko ON, Lin-Chao S. Escherichia coli responds to environmental changes using enolasic degradosomes and stabilized DicF sRNA to alter cellular morphology. Proceedings of the National Academy of Sciences 2017; 114:E8025-E34.
23. Tétart F, Bouché JP. Regulation of the expression of the cell-cycle gene ftsZ by DicF antisense RNA. Division does not require a fixed number of FtsZ molecules. Molecular microbiology 1992; 6:615-20.
24. Kittle J, Simons RW, Lee J, Kleckner N. Insertion sequence IS10 anti-sense pairing initiates by an interaction between the 5′ end of the target RNA and a loop in the anti-sense RNA. Journal of molecular biology 1989; 210:561-72.
25. Liu JM, Livny J, Lawrence MS, Kimball MD, Waldor MK, Camilli A. Experimental discovery of sRNAs in Vibrio cholerae by direct cloning, 5S/tRNA depletion and parallel sequencing. Nucleic acids research 2009; 37:e46-e.
26. Zhang Q, Zhang Y, Zhang X, Zhan L, Zhao X, Xu S, et al. The novel cis-encoded antisense RNA AsrC positively regulates the expression of rpoE-rseABC operon and thus enhances the motility of Salmonella enterica serovar typhi. Frontiers in microbiology 2015; 6:990.
27. Sharma CM, Hoffmann S, Darfeuille F, Reignier J, Findeiß S, Sittka A, et al. The primary transcriptome of the major human pathogen Helicobacter pylori. Nature 2010; 464:250-5.
28. Mandin P, Repoila F, Vergassola M, Geissmann T, Cossart P. Identification of new noncoding RNAs in Listeria monocytogenes and prediction of mRNA targets. Nucleic acids research 2007; 35:962-74.
29. Gerhart E, Wagner H, Nordström K. Structural analysis of an RNA molecule involved in replication control of plasmid Rl. Nucleic acids research 1986; 14:2523-38.
30. Jiang X, Liu X, Law CO, Wang Y, Lo WU, Weng X, et al. The CTX-M-14 plasmid pHK01 encodes novel small RNAs and influences host growth and motility. FEMS Microbiology Ecology 2017; 93.
31. Light J, Molin S. Post-transcriptional control of expression of the repA gene of plasmid R1 mediated by a small RNA molecule. The EMBO journal 1983; 2:93-8.
32. Nordgren S, Slagter-Jäger JG, Wagner EGH. Real time kinetic studies of the interaction between folded antisense and target RNAs using surface plasmon resonance. Journal of molecular biology 2001; 310:1125-34.
33. Weinberg Z, Wang JX, Bogue J, Yang J, Corbino K, Moy RH, et al. Comparative genomics reveals 104 candidate structured RNAs from bacteria, archaea, and their metagenomes. Genome biology 2010; 11:1-17.
34. Datta N, Kontomichalou P. Penicillinase synthesis controlled by infectious R factors in Enterobacteriaceae. Nature 1965; 208:239-41.
35. Sayers EW, Barrett T, Benson DA, Bolton E, Bryant SH, Canese K, et al. Database resources of the national center for biotechnology information. Nucleic acids research 2010; 39:D38-D51.
36. Wickham H. Elegant graphics for data analysis. Media 2009; 35:10.1007.
37. Kluyver T, Ragan-Kelley B, Pérez F, Granger BE, Bussonnier M, Frederic J, et al. Jupyter Notebooks-a publishing format for reproducible computational workflows. 2016.
